# Weekend Hospital Admission and Outcomes Following Emergency Cholecystectomy: A National Analysis of 194,787 Admissions, 2018–2022

**DOI:** 10.3390/healthcare14142193

**Published:** 2026-07-20

**Authors:** Wael Alkattan

**Affiliations:** College of Medicine, Alfaisal University, Riyadh 11533, Saudi Arabia; walkattan@alfaisal.edu

**Keywords:** cholecystectomy, weekend effect, emergency surgery, National Inpatient Sample, mortality, length of stay, sepsis, hospital volume, administrative database

## Abstract

**Background**: Acute cholecystitis affects approximately 200,000 hospitalizations annually in the United States, and emergency cholecystectomy is its definitive treatment. Whether weekend hospital admission, which is often associated with reduced staffing and diagnostic resources, adversely affects surgical outcomes in this population is unclear. We evaluated the association between weekend hospital admission and in-hospital mortality and key secondary outcomes following emergency cholecystectomy. **Methods**: This retrospective cohort study used the National Inpatient Sample (NIS) from January 2018 through December 2022. Adults (≥18 years) with acute cholecystitis (identified by ICD-10-CM codes) who underwent nonelective cholecystectomy (identified by ICD-10-PCS codes) were included; elective admissions, pediatric patients, and admissions with missing discharge weights were excluded. The analytic cohort comprised 194,787 unweighted admissions, representing a weighted national estimate of approximately 973,935 hospitalizations. Weekend admission was defined as admission on Saturday or Sunday (vs. Monday through Friday). The primary outcome was in-hospital mortality. Secondary outcomes, defined from secondary (not principal) diagnosis fields and interpreted as coded in-hospital diagnoses, included prolonged length of stay (>75th percentile), sepsis, surgical site infection, bile duct injury, venous thromboembolism, respiratory failure, acute kidney injury, blood transfusion, and cardiac complications. Multivariable logistic regression with hospital-year cluster-robust standard errors was used, adjusting for pre-exposure patient and hospital characteristics; the Benjamini–Hochberg false discovery rate was applied across secondary outcomes. **Results**: Among 194,787 admissions (weekday, 73.2%; weekend, 26.8%), in-hospital mortality was 0.62% in weekday and 0.53% in weekend patients. After adjustment, weekend admission was not associated with in-hospital mortality (aOR 0.87; 95% CI, 0.75–1.00; *p* = 0.06). Weekend admission was associated with modestly lower odds of prolonged length of stay (aOR 0.90; 95% CI, 0.88–0.93; *p* < 0.001). Weekend admission was not associated with sepsis (5.3% vs. 5.3%; aOR 1.01; 95% CI, 0.96–1.06; *p* = 0.65), respiratory failure (aOR 1.05; 95% CI, 1.00–1.10; *p* = 0.06), overall complications, or the remaining secondary outcomes after correction for multiple comparisons. Findings were consistent across design-based survey-weighted, inverse-probability-weighted, transfer-excluded, and principal-diagnosis sensitivity analyses. **Conclusions**: Weekend admission was not associated with higher in-hospital mortality or with a higher risk of sepsis following emergency cholecystectomy in the United States; weekend patients had modestly shorter hospital stays. These findings provide no evidence of higher coded in-hospital mortality or coded in-hospital complications among weekend admissions in contemporary US practice.

## 1. Introduction

Acute cholecystitis represents one of the most common indications for emergency abdominal surgery in the United States, contributing to hundreds of thousands of hospitalizations annually and imposing a significant burden on both patients and health care systems [1]. Laparoscopic cholecystectomy remains the definitive treatment of choice, supported by decades of evidence demonstrating superior outcomes compared with open approaches, including lower morbidity, shorter hospital stay, and reduced costs [2]. Contemporary guidelines, including the Tokyo Guidelines 2018 (TG18), advocate for early laparoscopic cholecystectomy within 72 h of symptom onset for appropriate surgical candidates, reflecting strong evidence that delayed surgery is associated with worse outcomes and increased costs [3,4,5,6,7].

The ‘weekend effect’, the phenomenon of increased mortality or adverse outcomes among patients admitted on weekends compared with weekdays, has been described across a variety of medical and surgical conditions [8,9]. Several proposed mechanisms include reduced staffing levels and nurse-to-patient ratios, limited availability of diagnostic and procedural resources, and the preferential admission of higher-acuity patients on weekends when elective care is curtailed [10,11]. In emergency general surgery, the presence and magnitude of the weekend effect remain controversial: while some national studies and systematic reviews have identified higher postoperative mortality associated with weekend surgical care [12,13,14,15], others have found no significant difference once case-mix severity is adequately adjusted [9,10]. Notably, hospital volume and perioperative infrastructure appear to moderate the weekend effect, with higher-volume and better-resourced institutions largely mitigating disparities in weekend outcomes [10,16].

Despite the frequency of emergency cholecystectomy and its time-sensitive nature, the relationship between weekend admission and outcomes in this specific procedure remains incompletely characterized. Prior studies have been limited by single-institution designs, short follow-up periods, restriction to pediatric populations, or lack of adjustment for hospital-level clustering and important confounders [17,18]. The extent to which weekend admission affects in-hospital mortality, infection risk, and resource utilization across diverse hospital types—including rural, urban nonteaching, and urban teaching centers—in a contemporary national cohort has not been systematically examined. We therefore conducted a nationally representative retrospective cohort study using the National Inpatient Sample (NIS) from 2018 through 2022 to determine whether weekend hospital admission is associated with in-hospital mortality and other adverse outcomes following emergency cholecystectomy, as well as to explore the moderating roles of hospital volume, teaching status, and the COVID-19 pandemic era.

## 2. Materials and Methods

### 2.1. Data Source

We utilized the National Inpatient Sample (NIS) from 2018 through 2022, a stratified probability sample of approximately 20% of all discharges from US community hospitals maintained by the Agency for Healthcare Research and Quality (AHRQ) as part of the Healthcare Cost and Utilization Project (HCUP) [19]. The NIS provides discharge-level data including patient demographics, diagnoses and procedure codes (ICD-10-CM and ICD-10-PCS), hospital characteristics, and discharge weights that allow national estimates to be derived. This study used de-identified, publicly available data; therefore, institutional review board approval was not required.

### 2.2. Study Population

**Figure 1:**
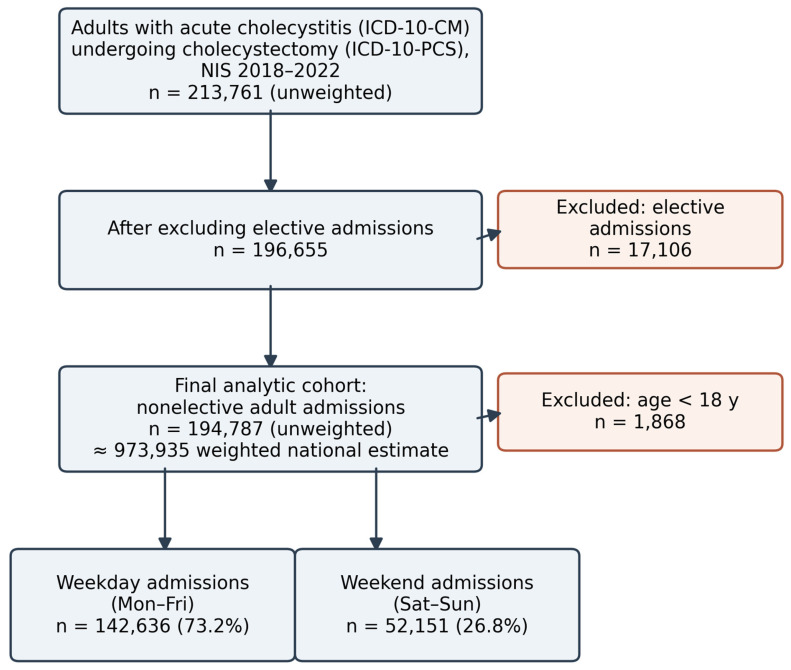
Cohort derivation (STROBE diagram).

We identified adult patients (≥18 years) with a principal or secondary diagnosis of acute cholecystitis using the following ICD-10-CM codes: K81.0 (acute cholecystitis), K80.00, K80.01, K80.12, K80.13, K80.42, K80.43, K80.62, K80.63, K80.66, and K80.67 (calculus with acute cholecystitis with or without obstruction, and acute-on-chronic variants). Cholecystectomy was identified using ICD-10-PCS codes for resection (0FT4) and excision (0FB4) of the gallbladder via open, laparoscopic, and endoscopic approaches; the complete code list and the algorithm for classifying laparoscopic, open, endoscopic, and conversion procedures are provided in Supplementary Table S1. We included only nonelective (emergency or urgent) admissions, identifying and excluding elective admissions using the NIS elective-admission indicator. Patients with a missing or invalid (non-positive) discharge weight were excluded. Of 213,761 admissions with acute cholecystitis undergoing cholecystectomy, 17,106 elective admissions and 1868 admissions of patients younger than 18 years were excluded, yielding a final analytic cohort of 194,787 nonelective adult admissions (Figure 1). This unweighted count is distinct from the corresponding weighted national estimate of approximately 973,935 hospitalizations; throughout, we report unweighted sample sizes for counts and discharge-weighted estimates for national rates.

### 2.3. Exposure

The primary exposure was weekend admission, defined as hospital admission on Saturday or Sunday. Weekday admissions (Monday through Friday) served as the reference group.

### 2.4. Outcomes

The primary outcome was in-hospital all-cause mortality. Secondary outcomes included: (1) prolonged length of stay, defined as length of stay exceeding the weighted 75th percentile of the cohort; (2) sepsis (ICD-10-CM codes A40, A41, R65.20, R65.21); (3) surgical site infection (T81.41-T81.43, T81.4xxA, K65 series); (4) bile duct injury (S36.13, K83.2); (5) venous thromboembolism (deep vein thrombosis and pulmonary embolism); (6) respiratory failure (J96, J80, J95 series); (7) acute kidney injury (AKI; N17 series); (8) blood transfusion; (9) cardiac complications (I21, I46, I49.0, I97 series); and (10) any in-hospital complication (composite of all preceding secondary outcomes). Admissions in which both laparoscopic and open cholecystectomy codes were recorded (n = 47) are described in Table 1 and Table 2 but were not modeled as an outcome, because administrative codes cannot distinguish true intraoperative conversion from staged or separately coded procedures. The ICD-10-CM code groups used to define these outcomes are listed in full in Supplementary Table S1, and their use in administrative data is supported by published validation studies of sepsis coding [20,21] and of Agency for Healthcare Research and Quality Patient Safety Indicator complication coding [22,23]. To reduce the misclassification of conditions present on admission as postoperative complications, complication codes were applied to secondary diagnosis fields only (diagnosis positions 2 through 40) and not to the principal diagnosis. The NIS for these years does not include a usable present-on-admission indicator [24]; accordingly, these outcomes are most appropriately interpreted as coded in-hospital diagnoses arising during admission rather than as definitively postoperative events.

### 2.5. Covariates and Comorbidities

Patient-level covariates included age (continuous), sex, race/ethnicity (White, Black, Hispanic, Asian/Pacific Islander, and other/unknown), primary payer (Medicare, Medicaid, private insurance, self-pay, and other), and median household income quartile by patient ZIP code. Hospital-level covariates, obtained from the NIS Hospital file linked by hospital identifier and year, included teaching status (rural, urban nonteaching, urban teaching), US Census region (Northeast, Midwest, South, West), hospital annual cholecystectomy volume (tercile: low, medium, high; thresholds defined from the full-period hospital volume distribution and held constant across years), and admission year. Comorbidity burden was measured using the van Walraven-weighted Elixhauser comorbidity index, derived from 31 comorbidity categories applied to all secondary diagnoses [25,26]. Transfer-in status was also included as a covariate.

### 2.6. Statistical Analysis

Baseline characteristics were compared between weekday and weekend admissions using standardized mean differences (SMDs), with an absolute SMD below 0.10 indicating negligible imbalance; because statistical significance is driven by the large sample size, we emphasize SMDs rather than *p*-values when interpreting baseline balance. For binary outcomes, we estimated adjusted odds ratios (aORs) and 95% confidence intervals (CIs) using multivariable generalized linear models with a binomial family and logit link, weighted by the NIS discharge weight, which was normalized to preserve the effective (unweighted) sample size so that national point estimates are obtained without spuriously inflating precision. Because the generalized linear model software we used does not implement full Taylor-series survey variance estimation, we accounted for the NIS sampling design by computing cluster-robust standard errors; standard errors were clustered on hospital-year units (unique hospital identifier within each NIS year; 14,976 clusters), because NIS hospital identifiers are not comparable across years. This approach approximates, rather than fully implements, the NIS complex survey design. To assess the impact of this approximation, we repeated the primary adjusted models in a fully design-based framework (Taylor-series linearization with NIS strata, hospital-year primary sampling units, and unnormalized discharge weights; R survey package), reported in Supplementary Table S10. Model diagnostics indicated no meaningful multicollinearity involving the exposure: the maximum variance inflation factor was 7.99, confined to dummy-coded levels of the multi-category race/ethnicity covariate (an expected consequence of coding mutually exclusive categories against a reference group), and the weekend-admission exposure itself had a variance inflation factor of 1.00. Missing values for categorical covariates were retained as a distinct category, and the number of missing observations for each key variable is reported in Supplementary Table S2. We pre-specified in-hospital mortality as the single primary outcome; prolonged length of stay, any complication, and cost as secondary outcomes; and the individual complications and all subgroup analyses as exploratory. For unadjusted comparisons of continuous outcomes, Mann–Whitney U tests were used. Benjamini–Hochberg false discovery rate adjustment was applied within the unadjusted (Table 2) and adjusted (Table 3) outcome families, and both nominal and FDR-adjusted *p*-values are shown in those tables. Subgroup, era, and sensitivity analyses (Table 4, Supplementary Tables S3, S4 and S6–S9) are exploratory and report nominal *p*-values only.

The primary multivariable model adjusted for pre-exposure covariates only (age, sex, race and ethnicity, primary payer, income quartile, Elixhauser comorbidity score, hospital teaching status, hospital region, transfer-in status, and year). Surgical approach and time-to-surgery occur after admission, may lie on the causal pathway between weekend admission and outcomes, and could act as mediators or colliders; they were therefore excluded from the primary model and examined only in a sensitivity analysis that additionally adjusted for them (Supplementary Table S3). As a primary sensitivity analysis, we used inverse probability of treatment weighting (IPTW). Stabilized weights were derived from a weighted logistic propensity score model on the full pre-exposure covariate set, truncated at the 1^st^ and 99^th^ percentiles, and combined multiplicatively with the NIS discharge weight; covariate balance was assessed by weighted standardized mean differences, and outcome models were weighted logistic regressions with hospital cluster-robust standard errors. A second sensitivity analysis excluded transferred-in patients (6.4% of weekday and 7.3% of weekend cases), and a third restricted the cohort to admissions with acute cholecystitis as the principal diagnosis (140,126 admissions) to address the possibility that broader cohort capture introduced selection bias (Supplementary Table S4). Prespecified, exploratory subgroup analyses were stratified by hospital annual cholecystectomy volume tercile (terciles defined from the distribution of hospital-level annual volume across the full study period, with thresholds held constant across years), hospital teaching status, and pandemic era (pre-COVID, 2018–2019 vs. COVID era, 2020–2022), with formal weekend-by-subgroup interaction terms estimated from pooled models. All analyses were performed in Python 3.14.4 (pandas 2.3.3, NumPy 2.4.4, statsmodels 0.14.6, SciPy 1.17.1, tableone 0.9.6), and the design-based survey sensitivity analysis was performed in R 4.3.2 with the survey package (version 4.4.2), with a two-sided *p* < 0.05 considered statistically significant. A step-by-step description of the analytic pipeline, software environment, and internal audit is provided in the File S1.

## 3. Results

### 3.1. Cohort Characteristics

A total of 194,787 nonelective adult admissions for cholecystectomy with acute cholecystitis met the inclusion criteria, of which 142,636 (73.2%) were weekday admissions and 52,151 (26.8%) were weekend admissions; this unweighted cohort corresponds to a weighted national estimate of approximately 973,935 hospitalizations. Admissions originated from 14,976 hospital-year units (2910 to 3065 hospitals contributing cases in each year from 2018 to 2022). Baseline characteristics are presented in Table 1, and the cohort derivation is shown in Figure 1. All baseline differences between weekday and weekend groups were small (all absolute SMDs < 0.10). The mean age was 55.5 ± 18.9 years in weekday and 55.4 ± 19.0 years in weekend patients (SMD 0.008). Female sex comprised 58.5% and 58.7% of weekday and weekend admissions, respectively. Comorbidity burden was similar between groups (mean Elixhauser van Walraven score 4.3 ± 7.6 vs. 4.2 ± 7.6; SMD 0.010). A median of 1.0 day elapsed from admission to surgery (measured in hospital days from the admission date) in both groups; 59.1% of weekday and 52.7% of weekend admissions underwent cholecystectomy by hospital day 1, that is, on the day of admission or the following hospital day (Supplementary Table S5).

**Figure 2:**
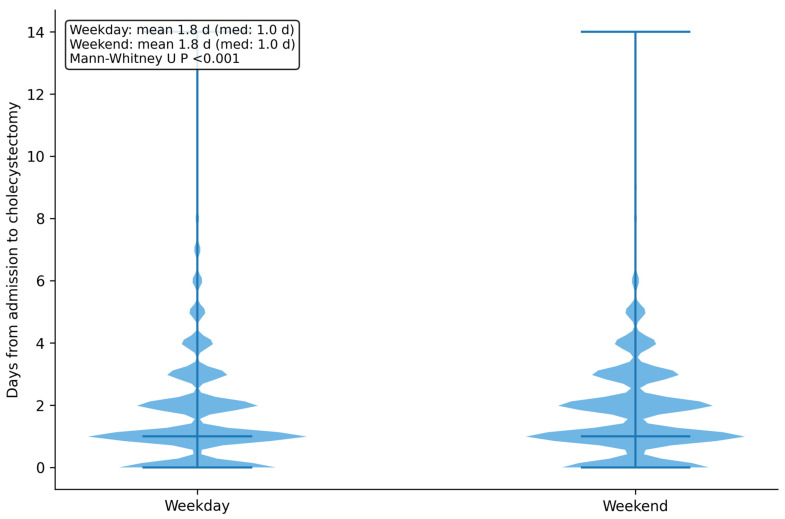
Distribution of time from admission to cholecystectomy, by admission day.

The laparoscopic approach was slightly more common among weekend patients (93.4% vs. 92.6%; SMD 0.032). Hospital teaching status and US Census region were well populated and similarly distributed between groups: most patients were treated at urban teaching hospitals (70.5% weekend vs. 69.7% weekday; rural, 6.9% vs. 7.4%; SMD 0.022), and the regional distribution (Northeast, Midwest, South, West) was nearly identical by admission day (SMD 0.014). Weekend patients were modestly more likely to be transferred in (7.3% vs. 6.4%). The distribution of time to cholecystectomy is shown in Figure 2.

### 3.2. Unadjusted Outcomes

Unadjusted outcome comparisons are presented in Table 2. In-hospital mortality was 0.62% among weekday patients and 0.53% among weekend patients (unadjusted OR 0.85; 95% CI, 0.74–0.97; *p* = 0.018). Prolonged length of stay occurred in 21.4% of weekday versus 19.9% of weekend patients (OR 0.91; 95% CI, 0.89–0.93; *p* < 0.001). Coded in-hospital sepsis was essentially identical between groups (5.3% vs. 5.3%; OR 1.00; 95% CI, 0.96–1.05; *p* = 0.97). No significant unadjusted differences were observed for respiratory failure, bile duct injury, venous thromboembolism, acute kidney injury, or overall complications after false discovery rate correction.

### 3.3. Adjusted Primary Analysis

**Figure 3:**
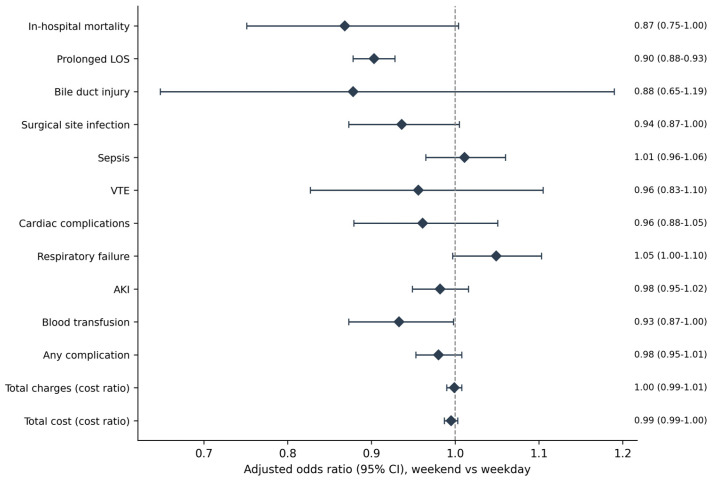
Adjusted odds ratios (95% CI) for clinical outcomes, weekend versus weekday admission (primary pre-exposure-adjusted model).

After multivariable adjustment for pre-exposure covariates (Table 3, Figure 3), weekend admission was not significantly associated with in-hospital mortality (aOR 0.868; 95% CI, 0.751–1.004; *p* = 0.056; 1166 events). Weekend admission was associated with modestly lower odds of prolonged length of stay (aOR 0.903; 95% CI, 0.878–0.928; *p* < 0.001; 40,869 events). It was not associated with sepsis (aOR 1.011; 95% CI, 0.965–1.060; *p* = 0.65; FDR-adjusted *p* = 0.70; 10,377 events) or respiratory failure (aOR 1.049; 95% CI, 0.997–1.103; *p* = 0.064; FDR-adjusted *p* = 0.18; 9014 events). No significant adjusted differences were found for overall complications (aOR 0.980; 95% CI, 0.953–1.008; *p* = 0.16), bile duct injury, surgical site infection, venous thromboembolism, acute kidney injury, cardiac complications, blood transfusion (aOR 0.933; 95% CI, 0.873–0.998; nominal *p* = 0.042 but FDR-adjusted *p* = 0.18). Total hospital cost did not differ by admission day (cost ratio 0.995; 95% CI, 0.987–1.003; *p* = 0.23).

### 3.4. Sensitivity Analyses

**Figure 4:**
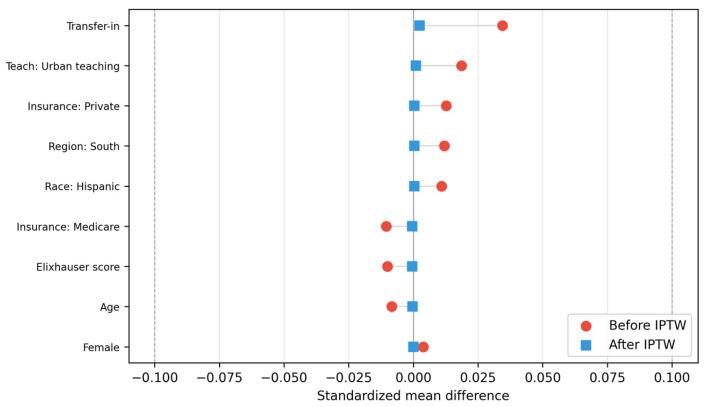
Covariate balance (standardized mean differences) before and after inverse probability of treatment weighting. Standardized mean differences shown for representative covariates; all covariates achieved post-weighting SMD below 0.10.

In inverse-probability-weighted (IPTW) analysis (Supplementary Table S6), covariate balance before and after weighting is shown in Figure 4; all post-weighting standardized mean differences were below 0.10. Findings were consistent with the primary analysis: weekend admission was not associated with in-hospital mortality (OR 0.869; 95% CI, 0.752–1.005; *p* = 0.06) or sepsis (OR 1.01; 95% CI, 0.96–1.06; *p* = 0.62), and remained associated with lower odds of prolonged length of stay (OR 0.903; 95% CI, 0.878–0.928; *p* < 0.001). After excluding transferred-in patients (Supplementary Table S7) and after restricting the cohort to admissions with acute cholecystitis as the principal diagnosis (Supplementary Table S4), results were materially unchanged, with the shorter length of stay among weekend patients persisting and no association observed for mortality or any coded complication. In the sensitivity analysis additionally adjusting for surgical approach and time-to-surgery (Supplementary Table S3), estimates for mortality, sepsis, and any coded complication were similar to the primary model, but respiratory failure showed a nominally significant association (aOR 1.07; 95% CI, 1.01–1.12; nominal *p* = 0.014, not adjusted for multiplicity). Prolonged length of stay was not modeled in this analysis because time-to-surgery is measured during the same hospitalization and is intrinsically related to length of stay. In the fully design-based survey analysis (Supplementary Table S10), estimates were materially unchanged (in-hospital mortality aOR 0.868, 95% CI 0.750–1.004; prolonged length of stay aOR 0.903, 95% CI 0.878–0.928), indicating that the cluster-robust approximation did not alter conclusions.

### 3.5. Subgroup Analyses

**Figure 5:**
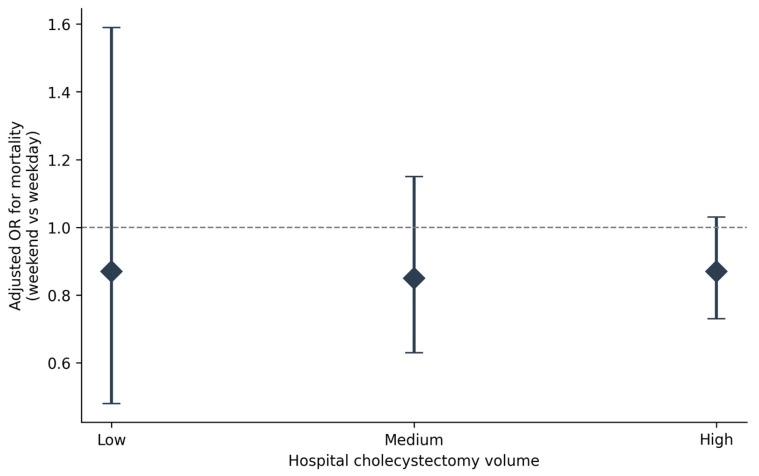
Weekend effect on in-hospital mortality by hospital cholecystectomy volume tercile.

Exploratory subgroup analyses were broadly consistent with the overall findings and are reported with formal interaction tests and false-discovery-rate caution (Supplementary Tables S8 and S9). When stratified by hospital annual cholecystectomy volume, stratum-specific estimates for prolonged length of stay were similar across hospital volume terciles, and the formal weekend-by-volume interaction was not statistically significant. Stratified analyses by hospital teaching status likewise showed no significant weekend-by-teaching interaction for mortality, prolonged length of stay, or overall complications. These subgroup analyses are vulnerable to sparse events and multiple testing and are therefore hypothesis-generating rather than confirmatory; we did not interpret individual stratum estimates as evidence of effect modification in the absence of a significant interaction term. Figure 5 depicts the volume-stratified estimates for in-hospital mortality graphically.

**Figure 6:**
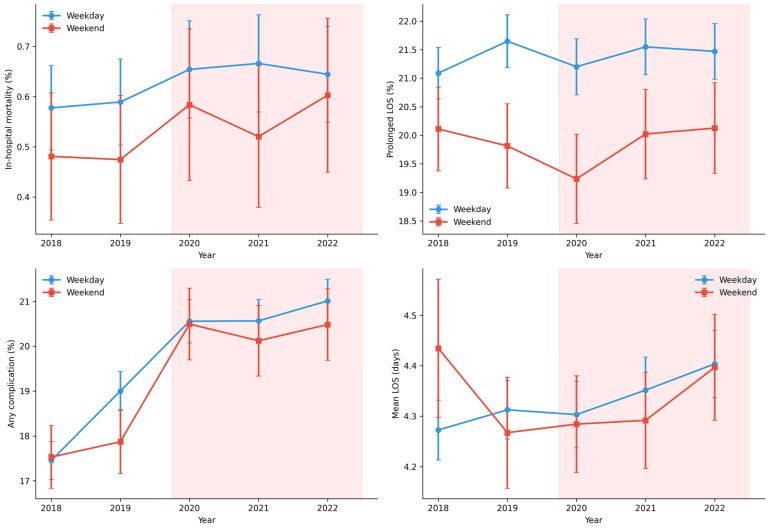
Temporal trends in study outcomes, 2018–2022, by admission day.

In era-stratified analyses (Table 4), no outcome showed a statistically significant weekend-by-era interaction (all interaction *p* > 0.20; smallest *p* = 0.237). Because era-stratified estimates derive from a single pooled interaction model rather than separate sparse models, no models failed to converge. Temporal trends in outcomes are displayed in Figure 6.

## 4. Discussion

In this nationally representative cohort of 194,787 nonelective emergency cholecystectomies from 2018 through 2022, weekend hospital admission was not associated with higher in-hospital mortality. Weekend patients demonstrated modestly lower odds of prolonged hospitalization; after restricting complication ascertainment to secondary diagnoses and adjusting for pre-exposure confounders, weekend admission was not associated with sepsis or respiratory failure. These results were consistent across inverse-probability-weighted, transfer-excluded, and principal-diagnosis sensitivity analyses, and the weekend effect was absent regardless of COVID-19 pandemic era. To our knowledge, this represents the largest national analysis of the weekend effect specifically in emergency cholecystectomy, encompassing five years of contemporary NIS data.

Our findings extend prior literature on the weekend effect in emergency surgery. A 2018 systematic review and meta-analysis by Smith et al. [9] found that postoperative mortality was higher after urgent or emergent weekend admissions; however, effect estimates were heterogeneous across procedures and were attenuated in registry-based studies with better case-mix adjustment. Hoehn et al. [12] specifically observed that among emergency general surgery procedures, cholecystectomy and appendectomy did not exhibit higher weekend mortality, in contrast to more complex operations such as partial colectomy and emergency laparotomy. Our adjusted analysis is consistent with these observations in a contemporary five-year cohort. The borderline result for mortality (aOR 0.868; *p*  =  0.056) is a non-significant trend and should not be over-read; selection effects that administrative data cannot capture remain a plausible explanation [10,16].

The paradoxically shorter length of stay and lower transfusion rates among weekend patients warrant careful interpretation. Weekend admissions were not operated on sooner: a smaller proportion of weekend than weekday admissions underwent cholecystectomy by hospital day 1 (52.7% vs. 59.1%; Supplementary Table S5), so earlier operative access does not explain the shorter stay. Although early definitive surgery for acute cholecystitis reduces total hospital stay and costs [5,6], our timing data argue against faster weekend surgery as the mechanism in this cohort. The shorter stay may instead reflect unmeasured differences in case mix, discharge practices, or coding, and we interpret it cautiously. The concentration of weekend cases in higher-volume urban hospitals with continuous emergency surgical coverage [10] is one hypothesis, but it is not directly testable in these data. In contrast to our initial analysis and to some prior reports, we did not find a higher rate of coded sepsis among weekend patients once present-on-admission and principal-diagnosis sepsis were excluded from the complication definition. This underscores that apparent weekend-sepsis associations in administrative data can arise from counting infections present at admission rather than hospital-acquired events and that proposed mechanisms such as reduced weekend staffing or delayed recognition of infectious complications [11] are not directly measurable in these data and remain speculative. Our findings therefore do not support sepsis-specific weekend quality-improvement mandates for this population. Although statistically significant, the association with prolonged length of stay was modest (aOR 0.90), and mean length of stay differed little between groups (4.3 vs. 4.3 days); this difference is of uncertain clinical importance for individual patients, although small per-admission differences may be relevant at the system level.

Stratified estimates for prolonged length of stay were similar across hospital volume terciles and teaching categories, and formal interaction tests showed no significant effect modification by volume, teaching status, or rural location (all interaction *p* ≥ 0.36). We therefore do not interpret stratum-specific significance or nonsignificance as evidence that the association differs across hospital types. Kothari et al. [10] reported that hospital perioperative capacity, including continuous operating room availability, intensivist coverage, and nursing staffing, can mitigate weekend effects in urgent general surgery; whether such factors operate in cholecystectomy care is a hypothesis that administrative data cannot test. Racial and insurance-related disparities in access to timely cholecystectomy are well documented [27,28,29] and remain important considerations for emergency surgical systems.

### Limitations

This study has several important limitations. First, as an administrative claims-based analysis, the NIS lacks granular clinical data including imaging findings, surgeon experience, precise operative timing, and postoperative medication administration, which may confound the observed associations. Second, unmeasured confounding cannot be excluded despite extensive covariate adjustment; although IPTW yielded consistent results, residual selection bias from unmeasured patient or hospital characteristics may remain. Third, the NIS does not allow tracking of individual patients across admissions, precluding analysis of 30-day readmission or long-term outcomes. Fourth, the complex survey design of the NIS was addressed primarily with hospital-year cluster-robust standard errors and normalized weights, which approximates the full design; a fully design-based re-analysis using NIS strata and unnormalized weights (Supplementary Table S10) produced materially identical estimates. Fifth, although the discharge weight was applied to all analyses, the analytic cohort included admissions from 2910 to 3065 hospitals in each NIS year (14,976 hospital-year units) and may not capture all US community hospitals; hospital region and teaching status were obtained by linkage to the year-specific NIS Hospital files, re-verified during the internal audit (File S1) [19]. Sixth, although complications were restricted to secondary diagnosis fields to reduce the misclassification of conditions present on admission, the NIS for these years lacks a usable present-on-admission indicator, so coded in-hospital diagnoses cannot be definitively classified as hospital-acquired; this is particularly relevant for sepsis and respiratory failure, which may reflect severity at presentation. Seventh, ICD-10-CM coding for complications may be subject to documentation variability across institutions, and these outcomes should be regarded as coded in-hospital diagnoses rather than adjudicated clinical events. Finally, despite extensive adjustment and consistent results across sensitivity analyses, residual and unmeasured confounding (for example, illness severity at presentation, surgeon experience, and time of day) cannot be excluded, and no causal interpretation should be attached to the associations reported here.

## 5. Conclusions

In this large national cohort of nonelective emergency cholecystectomies, weekend hospital admission was not associated with higher in-hospital mortality, and coded overall complication rates were similar between weekday and weekend admissions. After restricting complication ascertainment to secondary diagnoses and adjusting for pre-exposure confounders, weekend admission was not associated with sepsis or respiratory failure; the only consistent difference was a modestly shorter length of stay among weekend patients. These findings were robust across design-based, inverse-probability-weighted, transfer-excluded, and principal-diagnosis sensitivity analyses, and they provide no evidence of higher coded in-hospital mortality or coded in-hospital complications among weekend admissions in contemporary US practice. Given the limitations of administrative data, any residual differences in coded infectious diagnoses warrant confirmation in data sources with present-on-admission indicators and clinical detail before they are used to justify changes in weekend care processes.

## Figures and Tables

**Table 1:** Baseline characteristics of patients undergoing nonelective cholecystectomy by admission day (NIS 2018–2022).

Characteristic	Category	Overall	SMD	Weekday	Weekend	*p*-Value
n		194,787		142,636	52,151	
Age, mean (SD)		55.5 (18.9)	−0.008	55.5 (18.9)	55.4 (19.0)	0.105
Female sex, n (%)	0.0	80,736 (41.4)	0.005	59,193 (41.5)	21,543 (41.3)	0.677
Female sex, n (%)	1.0	114,037 (58.5)		83,432 (58.5)	30,605 (58.7)	
Female sex, n (%)	None	14 (0.0)		11 (0.0)	3 (0.0)	
Race/ethnicity, n (%)	Asian/PI	6508 (3.3)	0.018	4785 (3.4)	1723 (3.3)	0.065
Race/ethnicity, n (%)	Black	17,847 (9.2)		13,116 (9.2)	4731 (9.1)	
Race/ethnicity, n (%)	Hispanic	44,588 (22.9)		32,475 (22.8)	12,113 (23.2)	
Race/ethnicity, n (%)	Native American	1585 (0.8)		1188 (0.8)	397 (0.8)	
Race/ethnicity, n (%)	Other	7186 (3.7)		5311 (3.7)	1875 (3.6)	
Race/ethnicity, n (%)	Unknown	4151 (2.1)		2990 (2.1)	1161 (2.2)	
Race/ethnicity, n (%)	White	112,922 (58.0)		82,771 (58.0)	30,151 (57.8)	
Primary payer, n (%)	Medicaid	37,012 (19.0)	0.015	27,166 (19.0)	9846 (18.9)	0.217
Primary payer, n (%)	Medicare	71,969 (36.9)		52,895 (37.1)	19,074 (36.6)	
Primary payer, n (%)	No charge	1490 (0.8)		1076 (0.8)	414 (0.8)	
Primary payer, n (%)	Other	5546 (2.8)		4057 (2.8)	1489 (2.9)	
Primary payer, n (%)	Private	63,297 (32.5)		46,123 (32.3)	17,174 (32.9)	
Primary payer, n (%)	Self-pay	15,230 (7.8)		11,143 (7.8)	4087 (7.8)	
Primary payer, n (%)	Unknown	243 (0.1)		176 (0.1)	67 (0.1)	
Median household income quartile, n (%)	Q1 (lowest)	54,970 (28.2)	0.012	40,254 (28.2)	14,716 (28.2)	0.234
Median household income quartile, n (%)	Q2	50,289 (25.8)		36,791 (25.8)	13,498 (25.9)	
Median household income quartile, n (%)	Q3	48,004 (24.6)		35,300 (24.7)	12,704 (24.4)	
Median household income quartile, n (%)	Q4 (highest)	38,749 (19.9)		28,294 (19.8)	10,455 (20.0)	
Median household income quartile, n (%)	Unknown	2775 (1.4)		1997 (1.4)	778 (1.5)	
Elixhauser comorbidity score, mean (SD)		4.3 (7.6)	−0.010	4.3 (7.6)	4.2 (7.6)	0.050
Hospital teaching status, n (%)	Rural	14,090 (7.2)	0.022	10,508 (7.4)	3582 (6.9)	<0.001
Hospital teaching status, n (%)	Urban nonteaching	44,567 (22.9)		32,768 (23.0)	11,799 (22.6)	
Hospital teaching status, n (%)	Urban teaching	136,130 (69.9)		99,360 (69.7)	36,770 (70.5)	
US Census region, n (%)	Midwest	33,919 (17.4)	0.014	24,835 (17.4)	9084 (17.4)	0.072
US Census region, n (%)	Northeast	31,159 (16.0)		22,943 (16.1)	8216 (15.8)	
US Census region, n (%)	South	79,344 (40.7)		57,874 (40.6)	21,470 (41.2)	
US Census region, n (%)	West	50,365 (25.9)		36,984 (25.9)	13,381 (25.7)	
Transferred in, n (%)	0	181,864 (93.4)	0.034	133,505 (93.6)	48,359 (92.7)	<0.001
Transferred in, n (%)	1	12,923 (6.6)		9131 (6.4)	3792 (7.3)	
Surgical approach, n (%)	Laparoscopic	180,774 (92.8)	0.032	132,069 (92.6)	48,705 (93.4)	<0.001
Surgical approach, n (%)	Laparoscopic and open codes recorded	47 (0.0)		38 (0.0)	9 (0.0)	
Surgical approach, n (%)	Open	13,749 (7.1)		10,371 (7.3)	3378 (6.5)	
Surgical approach, n (%)	Other	217 (0.1)		158 (0.1)	59 (0.1)	
Time to surgery (days), median [Q1, Q3]		1.0 [1.0, 2.0]	0.034	1.0 [1.0, 2.0]	1.0 [1.0, 2.0]	<0.001
Length of stay (days), median [Q1, Q3]		3.0 [2.0, 5.0]	0.002	3.0 [2.0, 5.0]	3.0 [2.0, 5.0]	<0.001
Admission year, n (%)	2018.0	42,601 (21.9)	0.007	31,164 (21.8)	11,437 (21.9)	0.746
Admission year, n (%)	2019.0	41,718 (21.4)		30,549 (21.4)	11,169 (21.4)	
Admission year, n (%)	2020.0	36,361 (18.7)		26,598 (18.6)	9763 (18.7)	
Admission year, n (%)	2021.0	37,165 (19.1)		27,171 (19.0)	9994 (19.2)	
Admission year, n (%)	2022.0	36,942 (19.0)		27,154 (19.0)	9788 (18.8)	
Pandemic era, n (%)	COVID	110,468 (56.7)	0.002	80,923 (56.7)	29,545 (56.7)	0.753
Pandemic era, n (%)	Pre-COVID	84,319 (43.3)		61,713 (43.3)	22,606 (43.3)	
Congestive heart failure, n (%)	0	179,001 (91.9)	0.011	130,961 (91.8)	48,040 (92.1)	0.031
Congestive heart failure, n (%)	1	15,786 (8.1)		11,675 (8.2)	4111 (7.9)	
Cardiac arrhythmia, n (%)	0	166,657 (85.6)	0.004	122,085 (85.6)	44,572 (85.5)	0.492
Cardiac arrhythmia, n (%)	1	28,130 (14.4)		20,551 (14.4)	7579 (14.5)	
Diabetes, uncomplicated, n (%)	0	166,175 (85.3)	0.014	121,498 (85.2)	44,677 (85.7)	0.007
Diabetes, uncomplicated, n (%)	1	28,612 (14.7)		21,138 (14.8)	7474 (14.3)	
Diabetes, complicated, n (%)	0	179,705 (92.3)	0.012	131,475 (92.2)	48,230 (92.5)	0.026
Diabetes, complicated, n (%)	1	15,082 (7.7)		11,161 (7.8)	3921 (7.5)	
Renal failure, n (%)	0	176,006 (90.4)	0.010	128,774 (90.3)	47,232 (90.6)	0.059
Renal failure, n (%)	1	18,781 (9.6)		13,862 (9.7)	4919 (9.4)	
Liver disease, n (%)	0	169,561 (87.0)	0.006	124,090 (87.0)	45,471 (87.2)	0.264
Liver disease, n (%)	1	25,226 (13.0)		18,546 (13.0)	6680 (12.8)	
Obesity, n (%)	0	138,452 (71.1)	0.016	101,657 (71.3)	36,795 (70.6)	0.002
Obesity, n (%)	1	56,335 (28.9)		40,979 (28.7)	15,356 (29.4)	
Chronic pulmonary disease, n (%)	0	168,126 (86.3)	0.011	122,974 (86.2)	45,152 (86.6)	0.039
Chronic pulmonary disease, n (%)	1	26,661 (13.7)		19,662 (13.8)	6999 (13.4)	
Coagulopathy, n (%)	0	185,408 (95.2)	0.009	135,846 (95.2)	49,562 (95.0)	0.064
Coagulopathy, n (%)	1	9379 (4.8)		6790 (4.8)	2589 (5.0)	
Fluid and electrolyte disorders, n (%)	0	139,452 (71.6)	0.008	102,254 (71.7)	37,198 (71.3)	0.119
Fluid and electrolyte disorders, n (%)	1	55,335 (28.4)		40,382 (28.3)	14,953 (28.7)	
Hypertension, uncomplicated, n (%)	0	122,437 (62.9)	0.002	89,628 (62.8)	32,809 (62.9)	0.767
Hypertension, uncomplicated, n (%)	1	72,350 (37.1)		53,008 (37.2)	19,342 (37.1)	
Hypertension, complicated, n (%)	0	169,087 (86.8)	0.009	123,696 (86.7)	45,391 (87.0)	0.069
Hypertension, complicated, n (%)	1	25,700 (13.2)		18,940 (13.3)	6760 (13.0)	

Counts, percentages, means, and standardized mean differences (SMDs) in this table are unweighted sample values; the installed tableone release does not support survey-weighted summary statistics. The unweighted analytic cohort of 194,787 admissions corresponds to a weighted national estimate of approximately 973,935 admissions. SMD = standardized mean difference; emphasis should be placed on SMDs rather than *p*-values given the large sample. *p*-values from chi-square or Mann–Whitney U tests as appropriate. Co-recorded laparoscopic and open codes cannot be interpreted as intraoperative conversion; this row is descriptive and the pattern was not modeled as an outcome.

**Table 2:** Unadjusted clinical outcomes by admission day.

Outcome	Weekday (%)	Weekend (%)	Unadjusted OR (95% CI)	*p*-Value	FDR-Adjusted *p*-Value
In-hospital mortality	0.62	0.53	0.85 (0.74–0.97)	0.018	0.056
Prolonged LOS (>P75)	21.39	19.87	0.91 (0.89–0.93)	<0.001	<0.001
Length of stay (days)	4.3 (med: 3.0)	4.3 (med: 3.0)	—	<0.001	<0.001
Total charges ($)	82,343.1 (med: 60,999.0)	81,869.6 (med: 61,100.0)	—	0.563	0.600
Total cost ($)	18,051.1 (med: 14,240.5)	17,838.5 (med: 14,144.8)	—	0.066	0.133
Bile duct injury	0.12	0.11	0.88 (0.65–1.19)	0.409	0.497
Surgical site infection	2.26	2.11	0.93 (0.87–1.00)	0.051	0.117
Sepsis	5.33	5.33	1.00 (0.96–1.05)	0.969	0.969
VTE (DVT/PE)	0.51	0.48	0.94 (0.82–1.09)	0.435	0.497
Laparoscopic and open codes recorded	0.03	0.02	0.65 (0.31–1.35)	0.248	0.331
Cardiac complications	1.40	1.32	0.95 (0.87–1.04)	0.243	0.331
Respiratory failure	4.59	4.72	1.03 (0.98–1.08)	0.218	0.331
Acute kidney injury	11.94	11.68	0.98 (0.95–1.01)	0.114	0.202
Blood transfusion	2.64	2.43	0.92 (0.86–0.98)	0.011	0.043
Any complication	19.63	19.21	0.97 (0.95–1.00)	0.036	0.096
Time to surgery (days)	1.8 (med: 1.0)	1.8 (med: 1.0)	—	<0.001	<0.001

Weekday/weekend percentages and means (for continuous outcomes) are weighted using NIS discharge weights (DISCWT); medians are unweighted. OR = odds ratio, unadjusted (weekend admission only, no covariates), from weighted logistic regression with hospital-year cluster-robust 95% CI; CI = confidence interval. Continuous-outcome *p*-values are from an unweighted Mann–Whitney U test; binary-outcome *p*-values are from the weighted, cluster-robust logistic regression. Charges and cost reported as weighted mean (unweighted median, USD); cost estimated from charges using HCUP cost-to-charge ratios. Both nominal and Benjamini–Hochberg false-discovery-rate-adjusted *p*-values are shown. Co-recorded laparoscopic and open codes cannot be interpreted as intraoperative conversion; this row is descriptive and the pattern was not modeled as an outcome.

**Table 3:** Multivariable-adjusted odds ratios for clinical outcomes (weekend vs. weekday admission).

Outcome	Adjusted OR	95% CI	*p*-Value	FDR-Adjusted *p*-Value	N Events
In-hospital mortality	0.868	0.751–1.004	0.056	0.176	1166
Prolonged LOS	0.903	0.878–0.928	<0.001	<0.001	40,869
Bile duct injury	0.878	0.648–1.190	0.402	0.523	226
Surgical site infection	0.936	0.873–1.005	0.068	0.176	4319
Sepsis	1.011	0.965–1.060	0.648	0.702	10,377
VTE	0.956	0.827–1.105	0.543	0.642	970
Cardiac complications	0.961	0.879–1.051	0.386	0.523	2680
Respiratory failure	1.049	0.997–1.103	0.064	0.176	9014
AKI	0.982	0.949–1.016	0.297	0.483	23,118
Blood transfusion	0.933	0.873–0.998	0.042	0.176	5029
Any complication	0.980	0.953–1.008	0.164	0.355	38,021
Total charges (cost ratio)	0.999	0.990–1.008	0.842	0.842	193,812
Total cost (cost ratio)	0.995	0.987–1.003	0.229	0.426	193,812

Adjusted odds ratios (aOR) are from multivariable logistic regression with normalized NIS discharge weights and hospital-year cluster-robust standard errors (14,976 hospital-year clusters). Regression sample: 194,773 admissions (14 excluded for missing sex). N events are unweighted counts. Nominal and Benjamini–Hochberg false-discovery-rate (FDR)-adjusted *p*-values are both shown. Primary model adjusted for pre-exposure covariates only: age, sex, race, insurance, income, Elixhauser comorbidity score, hospital teaching status, hospital region, transfer status, and year. Surgical approach and time-to-surgery are post-exposure variables and are examined separately (Supplementary Table S3). Cost modeled via gamma generalized linear model with log link (reported as cost ratio).

**Table 4:** Weekend effect by pandemic era (pre-COVID vs. COVID) with formal interaction test.

Outcome	Pre-COVID aOR (95% CI)	COVID aOR (95% CI)	Interaction *p*-Value
In-hospital mortality	0.84 (0.67–1.06)	0.88 (0.73–1.06)	0.790
Prolonged LOS	0.91 (0.87–0.95)	0.90 (0.87–0.93)	0.658
Bile duct injury	0.84 (0.51–1.38)	0.90 (0.62–1.33)	0.821
Surgical site infection	0.91 (0.81–1.01)	0.96 (0.87–1.05)	0.442
Sepsis	1.04 (0.97–1.13)	0.99 (0.93–1.05)	0.268
VTE	1.00 (0.80–1.25)	0.93 (0.77–1.12)	0.640
Cardiac complications	1.02 (0.88–1.19)	0.92 (0.82–1.03)	0.282
Respiratory failure	1.02 (0.94–1.11)	1.06 (1.00–1.13)	0.430
AKI	0.98 (0.92–1.03)	0.98 (0.94–1.03)	0.829
Blood transfusion	0.89 (0.80–0.98)	0.96 (0.88–1.05)	0.237
Any complication	0.97 (0.92–1.01)	0.99 (0.95–1.02)	0.490

aOR = adjusted odds ratio, from weighted logistic regression (normalized NIS discharge weights) with hospital-year cluster-robust standard errors; covariates age, sex, Elixhauser score, transfer status, and region. Pre-COVID era: 2018–2019; COVID era: 2020–2022. The interaction *p*-value is the weekend × era term from a single pooled model (full sample); *p*-values are nominal and not adjusted for multiple comparisons; this analysis is exploratory.

## Data Availability

The data presented in this study are available from the Healthcare Cost and Utilization Project (HCUP), Agency for Healthcare Research and Quality. The National Inpatient Sample (NIS) is publicly available for purchase at https://www.hcup-us.ahrq.gov/. The complete analysis code (cohort construction, outcome definitions, models, and table and figure generation) is available from the corresponding author on reasonable request.

## Supplementary Materials

The following supporting information can be downloaded at: https://www.mdpi.com/article/10.3390/healthcare14142193/s1, Table S1: ICD-10-CM and ICD-10-PCS Code Dictionary and Algorithm; Table S2: Missing Observations for Key Variables; Table S3: Sensitivity Analysis Additionally Adjusting for Surgical Approach and Time-to-Surgery; Table S4: Sensitivity Analysis Restricted to Acute Cholecystitis as the Principal Diagnosis; Table S5: Time to Surgery and Proportion Operated by Hospital Day 1; Table S6: Inverse-Probability-of-Treatment-Weighted (IPTW) Outcomes (Weekend vs. Weekday Admission); Table S7: Sensitivity Analysis: Excluding Transferred-in Patients; Table S8: Hospital Volume Subgroup Analysis (Exploratory); Table S9: Hospital Teaching Status Subgroup Analysis (Exploratory); Table S10: Design-Based Survey-Weighted Sensitivity Analysis (Taylor-Series Linearization) for Weekend versus Weekday Admission; File S1: Data Pipeline, Software, and Internal Audit.

## Abbreviations

The following abbreviations are used in this manuscript:

NIS	National Inpatient Sample
HCUP	Healthcare Cost and Utilization Project
AHRQ	Agency for Healthcare Research and Quality
AKI	Acute Kidney Injury
ICD-10-CM	International Classification of Diseases, 10th Revision, Clinical Modification
ICD-10-PCS	International Classification of Diseases, 10th Revision, Procedure Coding System
aOR	Adjusted Odds Ratio
CI	Confidence Interval
LOS	Length of Stay
IPTW	Inverse Probability of Treatment Weighting
SMD	Standardized Mean Difference
IRB	Institutional Review Board
COVID-19	Coronavirus Disease 2019
FDR	False Discovery Rate
PSU	Primary Sampling Unit
