# Supplementary material for: Weekend Hospital Admission and Outcomes Following Emergency Cholecystectomy: A National Analysis of 194,787 Admissions, 2018–2022
Source: Healthcare (Basel). 2026 Jul 20;14(14):2193. doi: 10.3390/healthcare14142193 (PMC13411260; doi:10.3390/healthcare14142193)
Supplement: Supplementary file 1 [file healthcare-14-02193-s001.zip › File S1.pdf]

Supplementary Analytic Appendix: Data Pipeline, Software, and Internal Audit

1. Data Sources and Cohort Construction

This analysis used the Healthcare Cost and Utilization Project (HCUP) National Inpatient Sample (NIS) for data years 2018 through 2022, combining the Core, Hospital, and Cost-to-Charge Ratio files for each year. Acute cholecystitis was identified using ICD-10-CM diagnosis codes in any diagnosis position, and cholecystectomy was identified using ICD-10-PCS procedure codes; the complete code dictionary and case-finding algorithm are provided in Supplementary Table S1.

Admissions were required to be nonelective (emergency or urgent) and to carry a valid, positive discharge weight. Of 213,761 admissions initially matched on these diagnosis and procedure codes, 17,106 elective admissions and 1,868 admissions among patients younger than 18 years were excluded, leaving an analytic cohort of 194,787 admissions. Applying the NIS discharge weight (DISCWT), this unweighted cohort corresponds to a weighted national estimate of approximately 973,935 hospitalizations.

2. Variable Derivation

Weekend admission was defined from the NIS AWEEKEND indicator (Saturday or Sunday admission versus Monday through Friday). Hospital teaching status was taken from the NIS Hospital files (HOSP_LOCTEACH, coded 1 = rural, 2 = urban nonteaching, 3 = urban teaching) rather than from the corresponding field in the Core file; the fixed-width offset used to parse hospital teaching status from the Core file was found to read an unrelated position in the record layout and was not used for any reported statistic. Hospital region and teaching status were merged into the analytic file by hospital identifier and data year.

Annual hospital cholecystectomy volume was computed as the count of eligible cases per hospital per data year (hospital-year), and hospitals were divided into low-, medium-, and high-volume terciles based on the distribution of hospital-year volumes. Complication outcomes (bile duct injury, surgical site infection, sepsis, venous thromboembolism, cardiac complications, respiratory failure, acute kidney injury, blood transfusion) were identified from secondary diagnosis and procedure fields only (diagnosis positions 2 through 40), excluding the principal diagnosis position, to reduce misclassification of conditions present on admission; the NIS for 2018-2022 does not include a usable present-on-admission indicator. Time to surgery was derived from PRDAY, the NIS procedure-day field, which records the hospital day on which a procedure was performed (admission day = hospital day 0) rather than a clock-time interval.

3. Statistical Models

The primary and subgroup models were weighted logistic regressions of each outcome on weekend admission, using NIS discharge weights normalized so that the sum of weights equaled the unweighted analytic sample size rather than the much larger weighted national estimate; this keeps the effective sample size, and therefore the width of confidence intervals, consistent with the number of admissions actually observed. Standard errors were estimated with a cluster-robust sandwich estimator, clustering on hospital-year (the combination of the NIS hospital identifier and data year) rather than on the hospital identifier alone, because NIS hospital identifiers are assigned within each data year and are not a stable identifier for the same hospital across years. This produced 14,976 hospital-year clusters, compared with 4,774 unique hospital identifiers if data year were disregarded.

The primary multivariable model (Table 3) adjusted for age, sex, race and ethnicity, primary payer, median household income quartile, Elixhauser comorbidity score, hospital teaching status, hospital region, transfer-in status, and admission year. The regression sample comprised 194,773 admissions; 14

admissions were excluded for missing sex. Benjamini-Hochberg false discovery rate (FDR) correction was applied within the unadjusted (Table 2) and adjusted (Table 3) outcome families, and both nominal and FDR-adjusted p-values are reported in those two tables. All subgroup, era-interaction, and sensitivity analyses (Table 4 and Supplementary Tables S3, S4, S6, S7, S8, S9) are exploratory and report nominal p-values only, without correction for multiple comparisons.

4. Sensitivity Analyses

The following sensitivity and subgroup analyses were performed. Inverse-probability-of-treatment weighting (IPTW; Supplementary Table S6) used a weighted logistic propensity model on the full pre-exposure covariate set, with stabilized weights truncated at the 1st and 99th percentiles and combined multiplicatively with the normalized discharge weight. A transfer-excluded analysis (Supplementary Table S7) restricted the primary model to direct admissions. A principal-diagnosis-restricted analysis (Supplementary Table S4) restricted the cohort to admissions with acute cholecystitis coded as the principal diagnosis. An approach-and-timing-adjusted analysis (Supplementary Table S3) added surgical approach and time-to-surgery to the primary covariate set, since both are post-exposure variables on the causal pathway; prolonged length of stay was excluded from this analysis because time-to-surgery, measured during the same hospitalization, is intrinsically related to length of stay, and missing time-to-surgery values (5.6% of the cohort) were median-imputed. A formal weekend-by-era interaction analysis (Table 4) tested whether the weekend effect differed between the pre-COVID-19 (2018-2019) and COVID-19 (2020-2022) periods.

Finally, a fully design-based survey-weighted analysis (Supplementary Table S10) re-fit the primary binary outcome models using Taylor-series linearization instead of cluster-robust sandwich estimation, with NIS stratum nested within year as the stratification variable (992 strata), hospital-year as the primary sampling unit (14,976 primary sampling units), and unnormalized NIS discharge weights; 27 strata (2.7%) contained a single primary sampling unit and were handled with a centered variance adjustment (options(survey.lonely.psu = 'adjust') in the R survey package). This analysis was implemented in R rather than Python and used the same covariate set and the same analytic sample (194,773 admissions) as the primary model.

5. Internal Audit Performed for This Revision

A structured internal audit of the analytic pipeline was performed for this revision. Hospital teaching-status coding was re-verified against the NIS Hospital files; the stratum identifiers produced by an independent parse of the Hospital files agreed with the values used in the analysis for 100% of hospital-year records, and the Core-file teaching-status column was confirmed to be unreliable and was not used for any reported statistic. The weekend-admission flag was verified to have zero missing values in the AWEEKEND field. The cohort-exclusion arithmetic (213,761 matched admissions; 17,106 elective and 1,868 pediatric exclusions; 194,787 analytic admissions) was independently re-verified.

The audit identified and corrected one substantive issue: the cluster variable used for robust standard errors had pooled hospital identifiers across NIS data years, understating the true number of independent hospital-year sampling units. Every cluster-robust model in the manuscript was re-run using the corrected hospital-year cluster variable. Point estimates and event counts were unchanged, no confidence bound moved by more than 0.002, and no conclusion changed; the largest p-value shift was for in-hospital mortality, which moved from 0.060 to 0.056 under the corrected clustering.

The audit also traced a defect in an earlier version of the approach-and-timing-adjusted sensitivity table (Supplementary Table S3), in which a degenerate model fit for prolonged length of stay had rendered as a non-informative placeholder. The cause was structural: adjusting a length-of-stay outcome for a covariate (time-to-surgery) measured during the same hospitalization is not an appropriate specification, and this outcome was removed from that sensitivity analysis rather than re-fit. Independent re-derivation of all

outcome variables directly from raw diagnosis and procedure codes produced zero mismatches against the analytic dataset. Finally, the design-based survey re-analysis (Supplementary Table S10) reproduced all primary model point estimates to within 0.0005, supporting the cluster-robust approximation used in the primary analysis.

6. Software

All analyses other than the design-based survey re-analysis were conducted in Python 3.14.4. The design-based survey re-analysis (Supplementary Table S10) was conducted in R 4.3.2 using the survey package. Package versions are listed in the table below. Analysis code is available from the corresponding author on reasonable request.

Supplementary Appendix Table. Software and Package Versions Used in the Analytic Pipeline

Software / Package	Version	Role
Python	3.14.4	Primary analysis environment
pandas	2.3.3	Data handling
NumPy	2.4.4	Numerical computation
statsmodels	0.14.6	Weighted GLM / cluster-robust regression
SciPy	1.17.1	Statistical tests (e.g., Mann-Whitney U)
tableone	0.9.6	Table 1 (unweighted; see Table 1 footnote)
matplotlib	3.10.8	Figures, including Figure 4 (Love plot)
pyarrow	24.0.0	Parquet I/O for cached analytic cohort
R	4.3.2	Design-based survey analysis (Supplementary Table S10)
survey (R package)	4.4.2	Taylor-series linearization survey design
