# Supplementary material for: Weekend Hospital Admission and Outcomes Following Emergency Cholecystectomy: A National Analysis of 194,787 Admissions, 2018–2022
Source: Healthcare (Basel). 2026 Jul 20;14(14):2193. doi: 10.3390/healthcare14142193 (PMC13411260; doi:10.3390/healthcare14142193)
Supplement: Supplementary file 1 [file healthcare-14-02193-s001.zip › TableS10_Design_Based (1).pdf]

Supplementary Table S10. Design-Based Survey-Weighted Sensitivity Analysis (Taylor-Series Linearization) for Weekend versus Weekday Admission.

Outcome	aOR	95% CI	p-value	N events
In-hospital mortality	0.87	0.75–1.00	0.056	1166
Prolonged LOS	0.90	0.88–0.93	<0.001	40869
Bile duct injury	0.88	0.65–1.19	0.402	226
Surgical site infection	0.94	0.87–1.00	0.067	4319
Sepsis	1.01	0.96–1.06	0.649	10377
VTE	0.96	0.83–1.11	0.543	970
Cardiac complications	0.96	0.88–1.05	0.386	2680
Respiratory failure	1.05	1.00–1.10	0.065	9014
AKI	0.98	0.95–1.02	0.297	23118
Blood transfusion	0.93	0.87–1.00	0.041	5029
Any complication	0.98	0.95–1.01	0.164	38021

Design-based estimates using NIS strata within year (992 strata), hospital-year primary sampling units (14,976), unnormalized discharge weights, and Taylor-series linearization (R survey package 4.4.2, quasibinomial svyglm); single-PSU strata (n = 27) handled by centered adjustment (options(survey.lonely.psu = 'adjust')); model sample 194,773; covariates identical to Table 3. P-values are nominal and not adjusted for multiple comparisons; this analysis mirrors the primary models in Table 3 as a sensitivity analysis.
