# Supplementary material for: Weekend Hospital Admission and Outcomes Following Emergency Cholecystectomy: A National Analysis of 194,787 Admissions, 2018–2022
Source: Healthcare (Basel). 2026 Jul 20;14(14):2193. doi: 10.3390/healthcare14142193 (PMC13411260; doi:10.3390/healthcare14142193)
Supplement: Supplementary file 1 [file healthcare-14-02193-s001.zip › TableS1_Coding_Dictionary.pdf]

Supplementary Table S1. ICD-10-CM and ICD-10-PCS Code Dictionary and Algorithm

Variable / outcome	Codes / definition
Cohort: acute cholecystitis (ICD-10-CM, any diagnosis position)	K81.0; K80.00, K80.01, K80.12, K80.13, K80.42, K80.43, K80.62, K80.63, K80.66, K80.67
Cholecystectomy procedure (ICD-10-PCS)	Resection 0FT40ZZ, 0FT44ZZ, 0FT47ZZ, 0FT48ZZ; Excision 0FB40ZZ, 0FB44ZZ, 0FB47ZZ, 0FB48ZZ
Laparoscopic	0FT44ZZ, 0FB44ZZ, 0FT48ZZ, 0FB48ZZ (percutaneous endoscopic / via natural or artificial opening endoscopic)
Open	0FT40ZZ, 0FB40ZZ
Endoscopic / via opening	0FT47ZZ, 0FB47ZZ
Laparoscopic and open codes recorded (descriptive only; not modeled)	An open code (0FT40ZZ or 0FB40ZZ) recorded with a laparoscopic code in the same admission (rare; see limitations)
Exposure: weekend admission	Admission on Saturday or Sunday (vs Monday-Friday)
Eligibility	Adults >=18 y; nonelective (emergency or urgent) admissions; valid (positive) discharge weight
Complications (secondary diagnosis positions 2-40 only):	
Sepsis	A40.x, A41.x, R65.20, R65.21
Surgical site infection	T81.41-T81.43, T81.4XXA, K65.x
Bile duct injury	S36.13x, K83.2
Venous thromboembolism	I82.4x (DVT), I26.x (PE)
Respiratory failure	J96.0x, J96.2x, J96.9x, J80, J95 series
Acute kidney injury	N17.x
Cardiac complications	I21.x, I46.x, I49.0, I97.x
Blood transfusion (ICD-10-PCS)	codes beginning 30233 (transfusion via peripheral vein)
Comorbidity index	Elixhauser/van Walraven (31 categories), secondary diagnoses

Complication codes were applied to secondary diagnosis fields (positions 2-40) only, not to the principal diagnosis, to reduce misclassification of conditions present on admission. The NIS for 2018-2022 does not include a usable present-on-admission indicator.
