# Supplementary material for: Weekend Hospital Admission and Outcomes Following Emergency Cholecystectomy: A National Analysis of 194,787 Admissions, 2018–2022
Source: Healthcare (Basel). 2026 Jul 20;14(14):2193. doi: 10.3390/healthcare14142193 (PMC13411260; doi:10.3390/healthcare14142193)
Supplement: Supplementary file 1 [file healthcare-14-02193-s001.zip › TableS2_Missingness.pdf]

Supplementary Table S2. Missing Observations for Key Variables

Variable	N missing	% missing
Age	0	0.0
Female sex	14	0.01
Race/ethnicity	4151	2.13
Primary payer	243	0.12
Median household income quartile	2775	1.42
Elixhauser comorbidity score	0	0.0
Hospital teaching status	0	0.0
US Census region	0	0.0
Transferred in	0	0.0
Surgical approach	0	0.0
Time to surgery (days)	10926	5.61
Length of stay (days)	2	0.0
Total charges	961	0.49
Estimated cost	961	0.49

Counts and percentages of missing or unknown values among the analytic cohort (N = 194,787) are unweighted.
