# Supplementary material for: Weekend Hospital Admission and Outcomes Following Emergency Cholecystectomy: A National Analysis of 194,787 Admissions, 2018–2022
Source: Healthcare (Basel). 2026 Jul 20;14(14):2193. doi: 10.3390/healthcare14142193 (PMC13411260; doi:10.3390/healthcare14142193)
Supplement: Supplementary file 1 [file healthcare-14-02193-s001.zip › TableS3_Approach_Adjusted.pdf]

Supplementary Table S3. Sensitivity Analysis Additionally Adjusting for Surgical Approach and Time-to-Surgery

Outcome	aOR (95% CI)	p-value	N events
In-hospital mortality	0.88 (0.76–1.03)	0.103	1166
Sepsis	1.02 (0.97–1.07)	0.372	10377
Respiratory failure	1.07 (1.01–1.12)	0.014	9014
Any complication	0.98 (0.95–1.01)	0.186	38021

aOR = adjusted odds ratio, from weighted logistic regression (normalized NIS discharge weights) with hospital-year cluster-robust standard errors. Identical to the primary model (Table 3) but additionally including surgical approach and time-to-surgery, which are post-exposure variables on the causal pathway; presented only as a sensitivity analysis. Prolonged length of stay was excluded from this sensitivity analysis because time-to-surgery is measured during the same hospitalization and is intrinsically related to length of stay; missing time-to-surgery values (5.6%) were median-imputed. P-values are nominal and not adjusted for multiple comparisons; this analysis is exploratory.
