# Supplementary material for: Weekend Hospital Admission and Outcomes Following Emergency Cholecystectomy: A National Analysis of 194,787 Admissions, 2018–2022
Source: Healthcare (Basel). 2026 Jul 20;14(14):2193. doi: 10.3390/healthcare14142193 (PMC13411260; doi:10.3390/healthcare14142193)
Supplement: Supplementary file 1 [file healthcare-14-02193-s001.zip › TableS4_Principal_Dx.pdf]

Supplementary Table S4. Sensitivity Analysis Restricted to Acute Cholecystitis as the Principal Diagnosis

Outcome	aOR (95% CI)	p-value	N events
In-hospital mortality	0.85 (0.65–1.11)	0.227	332
Prolonged LOS	0.90 (0.87–0.93)	<0.001	20907
Sepsis	1.02 (0.92–1.12)	0.749	2170
Respiratory failure	1.02 (0.95–1.10)	0.565	3668
Any complication	0.98 (0.94–1.02)	0.245	19089

aOR = adjusted odds ratio, from weighted logistic regression (normalized NIS discharge weights) with hospital-year cluster-robust standard errors; primary covariate set (as in Table 3). Cohort restricted to admissions with acute cholecystitis coded as the principal diagnosis. P-values are nominal and not adjusted for multiple comparisons; this analysis is exploratory.
