# Supplementary material for: Weekend Hospital Admission and Outcomes Following Emergency Cholecystectomy: A National Analysis of 194,787 Admissions, 2018–2022
Source: Healthcare (Basel). 2026 Jul 20;14(14):2193. doi: 10.3390/healthcare14142193 (PMC13411260; doi:10.3390/healthcare14142193)
Supplement: Supplementary file 1 [file healthcare-14-02193-s001.zip › TableS5_Timing.pdf]

Supplementary Table S5. Time to Surgery and Proportion Operated by Hospital Day 1

Group	N (unweighted)	Surgery by hospital day 1 (weighted %)	Median time to surgery (days)
Weekday	134563	59.1	1.0
Weekend	49298	52.7	1.0

Percentages are weighted using NIS discharge weights (DISCWT); medians are unweighted. Time to surgery is measured in hospital days from admission (admission day = hospital day 0). 'Operated by hospital day 1' denotes a procedure performed on hospital day 0 or day 1; the NIS records procedure timing in hospital days, not clock hours, so this is not a literal 24-hour cutoff. Restricted to admissions with recorded procedure timing (N = 183,861); 10,926 admissions (5.6%) with missing timing excluded.
