# Supplementary material for: Weekend Hospital Admission and Outcomes Following Emergency Cholecystectomy: A National Analysis of 194,787 Admissions, 2018–2022
Source: Healthcare (Basel). 2026 Jul 20;14(14):2193. doi: 10.3390/healthcare14142193 (PMC13411260; doi:10.3390/healthcare14142193)
Supplement: Supplementary file 1 [file healthcare-14-02193-s001.zip › TableS6_IPTW_Outcomes.pdf]

Supplementary Table S6. Inverse-Probability-of-Treatment-Weighted (IPTW) Outcomes (Weekend vs. Weekday Admission)

Outcome	Weekday (%)	Weekend (%)	OR (95% CI)	p-value
In-hospital mortality	0.62	0.54	0.869 (0.752–1.005)	0.0582
Prolonged LOS (>P75)	21.35	19.97	0.903 (0.878–0.928)	0.0000
Bile duct injury	0.12	0.11	0.876 (0.645–1.188)	0.3934
Surgical site infection	2.25	2.12	0.936 (0.873–1.005)	0.0678
Sepsis	5.31	5.37	1.012 (0.965–1.061)	0.6249
VTE (DVT/PE)	0.50	0.48	0.956 (0.827–1.106)	0.5454
Cardiac complications	1.39	1.33	0.961 (0.878–1.051)	0.3833
Respiratory failure	4.58	4.77	1.051 (0.999–1.105)	0.0536
Acute kidney injury	11.91	11.77	0.983 (0.949–1.017)	0.3146
Blood transfusion	2.63	2.46	0.934 (0.874–0.999)	0.0465
Any complication	19.59	19.34	0.981 (0.953–1.009)	0.1768

Weekday/Weekend percentages are weighted using the final combined weight (stabilized IPTW × normalized NIS discharge weight). Stabilized inverse-probability-of-treatment weights (IPTW) were derived from a weighted logistic propensity score (truncated at the 1st/99th percentiles) and combined multiplicatively with the normalized NIS discharge weight. Outcome models are weighted logistic regressions with hospital-year cluster-robust standard errors (CLUSTER_ID = HOSP_NIS × YEAR; HOSP_NIS alone is not a stable hospital identifier across NIS years). OR = odds ratio; CI = confidence interval. P-values are nominal and not adjusted for multiple comparisons; this analysis is exploratory.
