# Supplementary material for: Weekend Hospital Admission and Outcomes Following Emergency Cholecystectomy: A National Analysis of 194,787 Admissions, 2018–2022
Source: Healthcare (Basel). 2026 Jul 20;14(14):2193. doi: 10.3390/healthcare14142193 (PMC13411260; doi:10.3390/healthcare14142193)
Supplement: Supplementary file 1 [file healthcare-14-02193-s001.zip › TableS7_Sensitivity.pdf]

Supplementary Table S7. Sensitivity Analysis: Excluding Transferred-in Patients

Analysis	Outcome	aOR (95% CI)	p-value
Exclude transfers	Mortality	0.905 (0.776–1.056)	0.204
Exclude transfers	Prolonged LOS	0.902 (0.876–0.928)	<0.001
Exclude transfers	Any complication	0.987 (0.958–1.016)	0.364

aOR = adjusted odds ratio, from weighted logistic regression (normalized NIS discharge weights) with hospital-year cluster-robust standard errors; same covariate set as the primary model (Table 3), excluding transfer status. Analysis restricted to direct admissions (transferred-in patients excluded). P-values are nominal and not adjusted for multiple comparisons; this analysis is exploratory.
