# Supplementary material for: Weekend Hospital Admission and Outcomes Following Emergency Cholecystectomy: A National Analysis of 194,787 Admissions, 2018–2022
Source: Healthcare (Basel). 2026 Jul 20;14(14):2193. doi: 10.3390/healthcare14142193 (PMC13411260; doi:10.3390/healthcare14142193)
Supplement: Supplementary file 1 [file healthcare-14-02193-s001.zip › TableS8_Volume_Subgroup.pdf]

Supplementary Table S8. Hospital Volume Subgroup Analysis (Exploratory)

Outcome	Volume	aOR (95% CI)	p-value
Mortality	Low	0.87 (0.48–1.59)	0.653
Mortality	Medium	0.85 (0.63–1.15)	0.286
Mortality	High	0.87 (0.73–1.03)	0.105
Mortality	Weekend x High-volume interaction	1.00 (0.73–1.39)	0.977
Prolonged LOS	Low	0.91 (0.81–1.03)	0.139
Prolonged LOS	Medium	0.91 (0.86–0.96)	0.001
Prolonged LOS	High	0.90 (0.87–0.93)	<0.001
Prolonged LOS	Weekend x High-volume interaction	0.99 (0.93–1.05)	0.661
Any complication	Low	0.94 (0.84–1.06)	0.324
Any complication	Medium	0.96 (0.91–1.02)	0.187
Any complication	High	0.99 (0.95–1.02)	0.401
Any complication	Weekend x High-volume interaction	1.03 (0.97–1.10)	0.365

aOR = adjusted odds ratio, from weighted logistic regression (normalized NIS discharge weights) with hospital-year cluster-robust standard errors; covariates age, sex, Elixhauser score, transfer status, and region. Volume tertiles by annual hospital cholecystectomy volume. P-values are nominal and not adjusted for multiple comparisons; this analysis is exploratory/hypothesis-generating. The formal weekend × high-volume interaction is reported in the final row of each outcome.
