# Supplementary material for: Weekend Hospital Admission and Outcomes Following Emergency Cholecystectomy: A National Analysis of 194,787 Admissions, 2018–2022
Source: Healthcare (Basel). 2026 Jul 20;14(14):2193. doi: 10.3390/healthcare14142193 (PMC13411260; doi:10.3390/healthcare14142193)
Supplement: Supplementary file 1 [file healthcare-14-02193-s001.zip › TableS9_Teaching_Subgroup.pdf]

Supplementary Table S9. Hospital Teaching Status Subgroup Analysis (Exploratory)

Outcome	Hospital Type	aOR (95% CI)	p-value
Mortality	Rural	0.91 (0.54–1.54)	0.727
Mortality	Urban nonteaching	0.88 (0.66–1.19)	0.415
Mortality	Urban teaching	0.85 (0.72–1.02)	0.076
Mortality	Weekend x rural interaction	1.07 (0.62–1.83)	0.817
Prolonged LOS	Rural	0.91 (0.82–1.01)	0.088
Prolonged LOS	Urban nonteaching	0.92 (0.86–0.97)	0.003
Prolonged LOS	Urban teaching	0.90 (0.87–0.93)	<0.001
Prolonged LOS	Weekend x rural interaction	1.01 (0.90–1.13)	0.875
Any complication	Rural	0.99 (0.89–1.10)	0.851
Any complication	Urban nonteaching	0.99 (0.93–1.05)	0.672
Any complication	Urban teaching	0.97 (0.94–1.01)	0.103
Any complication	Weekend x rural interaction	1.02 (0.91–1.13)	0.789

aOR = adjusted odds ratio, from weighted logistic regression (normalized NIS discharge weights) with hospital-year cluster-robust standard errors; covariates age, sex, Elixhauser score, transfer status, and region. Teaching status from the NIS Hospital file. P-values are nominal and not adjusted for multiple comparisons; this analysis is exploratory/hypothesis-generating. The formal weekend × rural interaction is reported in the final row of each outcome.
